# A new continuous glucose monitor for the diagnosis of gestational diabetes mellitus: a pilot study

**DOI:** 10.1186/s12884-023-05496-7

**Published:** 2023-03-18

**Authors:** Daria Di Filippo, Amanda Henry, Chloe Bell, Sarah Haynes, Melissa Han Yiin Chang, Justine Darling, Alec Welsh

**Affiliations:** 1 Discipline of Women’s Health, School of Clinical Medicine, University of New South Wales Sydney, Locked Bag 2000, Barker Street, Randwick, NSW 2031 Australia; 2 Department of Women’s and Children’s Health, St George Hospital, Gray St, Kogarah, NSW 2217 Australia; 3 Diabetes Clinic Royal Hospital for Women, Barker Street - Randwick, Randwick, NSW 2031 Australia; 4 Department of Maternal-Fetal Medicine, Royal Hospital for Women, Barker Street - Randwick, Randwick, NSW 2031 Australia

**Keywords:** Gestational diabetes mellitus, Oral glucose tolerance test, Diagnosis, Continuous glucose monitoring

## Abstract

**Background:**

Gestational Diabetes Mellitus (GDM) incidence and adverse outcomes have increased globally. The validity of the oral glucose tolerance test (OGTT) for GDM diagnosis has long been questioned, with no suitable substitute reported yet. Continuous Glucose Monitoring (CGM) is potentially a more acceptable and comprehensive test. The aim of this study was to assess the Freestyle Libre Pro 2 acceptability as a diagnostic test for GDM, then triangulating its results with OGTT results as well as risk factors and sonographic features of GDM.

**Methods:**

Women wore the CGM device for 7 days at 24–28 weeks, undergoing the OGTT before CGM removal. CGM/OGTT acceptability as well as GDM risk factors evaluation occurred via three online surveys. CGM distribution/variability/time in range parameters, combined in a CGM Score of Variability (CGMSV), were triangulated with OGTT results and a risk-factor-based Total Risk Score (TRS). In a subgroup, GDM ultrasound features (as modified Ultrasound Gestational Diabetes Score – m-UGDS) were also incorporated.

**Results:**

Of 107 women recruited, 87 (81%) were included: 74 (85%) with negative OGTT (NGT) and 13 (15%) positive (GDM). No significant difference was found between NGT and GDM in terms of demographics (apart from family history of diabetes mellitus), CGM parameters and perinatal outcomes. Women considered CGM significantly more acceptable than OGTT (81% versus 27% rating 5/5, *p* < 0.001).

Of the 55 NGT with triangulation data, 28 were considered ‘true negative’ (TRS concordant with OGTT and CGMSV): of these 4/5 evaluated at ultrasound had m-UGDS below the cut-off. Five women were considered ‘false negative’ (negative OGTT with both TRS and CGMSV above the respective cut-offs). Triangulation identified also six ‘false positive’ women (positive OGTT but TRS and CGM both below the cut-offs). Only one woman for each of the last two categories had m-UGDS evaluated, with discordant results.

**Conclusions:**

CGM represents a more acceptable alternative for GDM diagnosis to the OGTT. CGM triangulation analysis suggests OGTT screening may result in both false positives and negatives. Further research including larger cohorts of patients, and additional triangulation elements (such as GDM biomarkers/outcomes and expanded m-UGDS) is needed to explore CGM potential for GDM diagnosis.

**Supplementary Information:**

The online version contains supplementary material available at 10.1186/s12884-023-05496-7.

## Background

Gestational Diabetes Mellitus (GDM) is a major public health issue, with steeply increasing incidence in the last decades due to a combination of maternal and environmental factors as well as changes in diagnostic strategies [1, 2]. Maternal and neonatal outcomes continue to be deeply impacted by this condition in both the short and long term, contributing to the current obesity and type 2 diabetes (T2DM) pandemic [2–4].

Although diagnostic thresholds and exact methods have varied widely over time, among different countries and even among different organizations within the same country, the current ‘gold standard’ for GDM diagnosis is still the oral glucose tolerance test (OGTT). However, an extensive list of pre-analytical (encompassing pre-testing (including preparatory diet and time of fasting, glucose load, collection tubes) and physiological factors (e.g. hydration, stress levels), as well as analytical (e.g. traceability/bias) and post-analytical limitations (results reporting and interpretation) have been reported for the OGTT [1, 5].

A number of potential substitutes for the OGTT have been proposed in the literature although none has been yet reported as a sufficiently robust candidate [6]. Of promise is Continuous Glucose Monitoring (CGM), which allows for evaluation of interstitial glucose levels during up to 14 days of ordinary life, as opposed to a one-off response to an artificial glucose load, offering a completely new perspective on glucose homeostasis [7]. To date, most research on CGM use in pregnancy has been regarding the management of type 1 diabetes (T1DM) and GDM, with little data on its application to GDM diagnosis [1, 8–10]. Our previous work demonstrates good acceptability of CGM as a diagnostic test for GDM, and its potential to unmask OGTT misdiagnosis [11].

The main limitation to developing a CGM-based diagnostic test for GDM is the lack of a gold standard aside from the deeply flawed OGTT. A solution to this issue may be provided by the concept of triangulation, which consists in evaluating an object from different perspectives to identify overlapping areas which represent the base for a new definition of the object [12]. Therefore, the aim of this study was to trial the Freestyle Libre Pro 2 as a diagnostic test for GDM, assessing its acceptability as opposed to the OGTT for a general population of pregnant women, and triangulating its results with risk factors and sonographic features of GDM as well as with the OGTT results.

## Methods

### Study design

This prospective cohort study was held in two metropolitan hospitals in Sydney between April 2021 and April 2022; delays occurred secondary to the Sydney lock-down in response to the Covid-19 pandemic, resulting in 3 months suspension of recruitment. Women enrolled in the antenatal care clinics of the two hospitals were eligible and were invited to participate in the study via SMS/phone calls. Exclusion criteria were pre-existing T1DM or T2DM, first/early second trimester diagnosis of GDM and mental illness precluding informed consent.

Interested women had the opportunity to clarify further details over the phone or during the first study appointment. After signing the consent form, the Freestyle Libre iPro 2 CGM monitor was applied on the back of the participant’s upper arm [13]. The application side was decided by each participant depending on preferred side for sleeping, writing and carrying heavy loads. Using the PRO version, participants were blinded to their CGM data and were then asked to download a free app to keep track of their diet and exercise sessions [14]. After 7 days of CGM wearing, each participant’s routine OGTT was undertaken by study staff at the recruitment site before having the sensor removed. The OGTT was performed using a 75 g glucose beverage and interpreted against the IADPSG criteria [15].

Prior to completion of their participation, women were requested to share their diet/physical activity diary from their app through an email, and to complete three questionnaires: one on their risk factors for GDM, one on the acceptability of OGTT and one on the acceptability of CGM (Additional files 1 and 2). Participants could find more information on the study and the link to the questionnaire on the study website www.cgm4gdm.net.

Regarding sample size, we aimed to recruit similar numbers of patients to our previous study using the Medtronic iPro 2, in order to compare pilot results between the two devices. Our previous study’s combined dropout/non-usable data rate was 39%, with reasons including poor compliance with food intake recording and finger pricks, as well as incomplete OGTT results [11]. Given that the Freestyle Libre PRO does not require finger pick calibration and that the OGTT was offered at the recruitment sites as part of this study, a lower dropout/data exclusion rate was anticipated. To allow data comparison, the recruitment goal was set at 100 women, accounting for a 20% dropout/data exclusion rate. Rates of negative outcomes (macrosomia defined as birthweight > 4 kg, preterm delivery < 37 weeks’ gestation, respiratory distress and elective/emergency caesarean sections) in the results of the NGT group in this study were used together with their OR described in a recent meta-analysis in GDM women to calculate the sample size needed to explore their correlation with CGM data in future using online software package “Select Statistical Service” [16, 17].

In a previous study, our group developed a survey for extensive evaluation of well-established and recently proposed risk factors for GDM, including data from 21 participants of this current pilot study [12]. Questions were on ethnicity, BMI, medical history (obstetric inclusive) but also exercise and dietary patterns, season of conception and ART use. We additionally created surveys on OGTT and CGM consisting of five questions regarding the overall acceptability as well as acceptability of insertion, wearing and removal, and the likelihood of recommending CGM as a diagnostic test for other women in a Likert scale format of 0–5. A final free text box allowed participants to share any recommendation or comment. The survey on CGM acceptability is the same used in our previous Medtronic pilot study to allow comparison [11].

### Data collection and analysis

Data collection and synthesis were based on the protocols of our previous studies on the use of the Medtronic iPro2 for GDM diagnosis and the development of a questionnaire for GDM risk factors [11, 12]. Clinical data was obtained from the hospitals’ obstetric database (Additional file 3). Cases were followed until birth.

Data from the Freestyle Libre PRO was downloaded using the web-based software portal (LibreView, app) and exported for analysis [18]. Glycaemic reports generated for each patient in Microsoft Excel were individually considered to determine validity for analysis. Only CGM output with 96 measurements per day for seven days were considered valid and analysed. Daytime was considered from 06:00 am to 23:59 h and night-time from 00.00 am to 05:59am. The CGM parameters considered in our analysis are outlined in Table 1.

**Table 1:** Continuous glucose monitor parameters used for data analysis

Sigle – Name	Definition/cut-off (reference)
Mean	Mean of blood glucose level registered at CGM [19]
SD – Standard deviation	Dispersion of the dataset relative to its mean [19]
CV – Coefficient variation	Mean corrected for SD (SD/Mean) [20]
TIR – Time in range	3.5–7.8 mmol/L [16]
TBR – Time below range	= 3.0 – 3.4 mmol/L, 2 = < 3.0 mmol/L [16]
TAR – Time above range	> 7.8 mmol/L [16], 2 = > 10 mmol/L
MAGE – Mean amplitude of glycaemic excursion	Measure of intra-day glycaemic variability [19]
MODD – Mean of daily differences	Measure of inter-daily glycaemic variability [19]

Statistical analysis was performed using Microsoft Excel (Microsoft, WA, USA) and SPSS (SPSS Inc, IL, USA). Normally distributed continuous variables are presented as mean ± standard deviation (SD); non-normally distributed continuous variables are presented as median with interquartile range. Continuous variables were compared between groups using t-test (normally distributed) and Mann Whitney U test (non-normally distributed) as appropriate. Categorical variables are presented as percentages and were compared using Chi-Square or Fisher's exact test as appropriate. Values of *p* < 0.05 were considered statistically significant. Due to the pilot/exploratory nature of the study no statistical adjustments for multiple comparisons were made.

### Triangulation

Triangulation may help to explore the issue when one is attempting to introduce a new measure that can only be compared against a current flawed gold-standard test. In order to not have to solely rely on OGTT as comparison for CGM data (combined in a score of variability (CGMSV)), we additionally triangulated CGMSV with risk factors, combined in a Total Risk Score (TRS) as already described in our previous publications [11, 12]. In a subgroup of women, triangulation also included the evaluation of ultrasound features of GDM, combined in a modified version of the Ultrasound Gestational Diabetes Score (m-UGDS) [21].

The CGMSV was calculated based both on the first three days and the complete 7 days period of CGM wearing. The parameters considered were of glucose levels’: (a) distribution: mean, SD, coefficient of variation; (b) variability: MAGE(Mean Amplitude of Glycaemic Excursion) for intra-day variability, and MODD (Mean of Daily Differences) for inter-day variability; (c) percentage of time spent in the range recommended for pregnant women (3.5–7.8 mmol/L) [7, 22]. These values were calculated on Excel after downloading raw data from the CGM system. MAGE and MODD were calculated using the Easy GV software [23]. CGMSV was calculated as a sum of the normalised values of mean, SD, CV, MAGE, MODD, TBR, TAR.

To calculate the TRS, each alternative response to the risk factors questionnaire was allocated a value based on the odds ratio (OR) for likelihood of development of GDM, with the baseline risk being 1 for each risk factor in its absence (i.e. 1 = baseline, risk factor not present). A total risk score was then calculated as the sum of the values (normalised against the baseline) recorded for each answer [12]. The cut-off value for CGMSV and TRS was estimated by finding the midpoint of the sum of the highest value in the NGT women and the lowest in the GDM women as already described in our previous publications [11, 12].

In a subgroup of women (*n* = 25) the triangulation analysis included a sonographic score of GDM, based on a modified protocol of the UGDS (m-UGDS), which was found to be a promising indicator of GDM in a recent systematic review published by our group, when assessed against the WHO ASSURED criteria (affordable, sensitive, specific, user-friendly, rapid and robust, equipment-free and deliverable to end-users) [6]. The ultrasound was performed by the study sonographer during or after the CGM monitoring period (24–28 weeks), excluding the day of the OGTT. The m-UGDS consisted of six parameters: fetal adipose subcutaneous tissue, asymmetrical macrosomia, cardiac circumference, cardiac width, interventricular septum thickness, immature appearance of placenta. We did not include the sonographic features of the UGDS that were less used in the recent years due to conflicting evidence in the literature, namely: breathing movements, placental thickness and immature placental appearance [24–26]. For Fetal Subcutaneous Adipose Thickness (SCAT), measures were taken from the inner edge of skin to the outer aspect of the echogenic subcutaneous fat surrounding the abdomen at the level of the fetal kidneys (as per Perovic et al.) and at level of the abdominal circumference, to then calculate the mean value and increase reproducibility [21]. All the other variables were measured as described in the original protocol, of which we also adopted the cut-offs values [21, 27]. The cut-off of the m-UGDS was set as > 3.

## Results

**Fig. 1:**
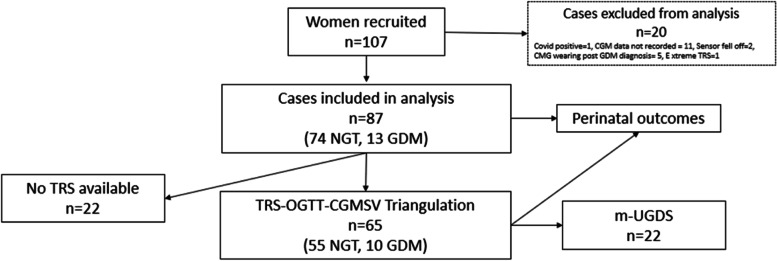
Consort diagram CGM = continuous glucose monitoring, TRS = total risk score, OGTT = oral glucose tolerance test, CGMSV = continuous glucose monitoring score of variability, m-UGDS = modified ultrasound gestational diabetes score

Of 107 women recruited to the study (Fig. 1), 87 were included (81%) in data analysis. The most common reason for exclusion was CGM data recording period < 7 days (*n* = 11). Four cases had less than 6 days recorded and seven had less than 100% coverage (96 readings) in the seventh day. Additionally, two cases had missing CGM data due to sensor misplacement.

Seventy-four participants (85%) had Normal Glucose Tolerance as per the OGTT (NGT group) and 13 (15%) were positive to the OGTT (GDM group). Triangulation was completed for 65 participants who completed the risk factor questionnaire enabling calculation of the TRS. Twenty-two of these patients also underwent an ultrasound for evaluating the m-UGDS. Perinatal outcomes were analysed for all included participants.

Maternal demographic characteristics are summarised in Table 2. Women classified as having GDM were significantly more likely to have a family history of diabetes mellitus (54% vs 23%, *p* = 0.03). All the OGTT values of time 0, 1 h and 2 h after the glucose load were significantly higher in the GDM group.

**Table 2:** Participant Demographic Characteristics

	**NGT (** ***n*** ** = 74)** **n (%)**	**GDM (** ***n*** ** = 13)** **n (%)**	***p*** **-value**
High Risk Background^a^	18 (24%)	5 (38%)	0.23
**Family History of DM**	**17 (23%)**	**7 (54%)**	**0.03**
Previous macrosomia	1 (3%)	1 (8%)	0.39
Previous GDM	3 (4%)	1 (8%)	0.48
Primiparity	40 (54%)	9 (75%)	0.15
	**Mean ± SD**	**Mean ± SD**	
Age	32.4 ( ±) 4.8	32.1 ( ±) 2.9	0.82
BMI	22.9 ( ±) 4.8	23.0 ( ±) 5.0	0.98
**OGTT time 0 (mmol/L)**	**4.3 ( ±) 0.3**	**4.6 ( ±) 0.6**	**0.01**
**OGTT 1 h (mmol/L)**	**6. 9 ( ±) 1.4**	**9.2 ( ±) 1.2**	** < 0.001**
**OGTT 2 h (mmol/L)**	**5. 7 ( ±) 1.2**	**8.1 ( ±) 1.7**	** < 0.001**

*GDM* Gestational diabetes mellitus, *NGT* normal glucose tolerance, *DM* Diabetes Mellitus, *SD* Standard deviation, *IR* interquartile range, *BMI* Body Mass Index

^a^High risk background = Southeast Asian, Chinese, Middle Eastern, Hispanic, South American, Aboriginal, Torres Strait Islander

Perinatal outcomes are described in Table 3. No significant difference was found in terms of perinatal outcomes for mothers and newborns in women classified by the OGTT as having NGT versus GDM. Of the 13 women diagnosed with GDM, 8 were managed with diet only and five with medication (one with insulin, one with oral hypoglycaemic agents and three with both insulin and oral hypoglycaemic agents).

**Table 3:** Perinatal outcomes in NGT versus GDM

	**NGT (** ***n*** ** = 74)** **n (%)**	**GDM (** ***n*** ** = 13)** **n (%)**	***p*** **-value**
Macrosomia suspected	4 (5%)	2 (15%)	0.22
Induction of labour	21 (29%)	5 (39%)	0.34
Second degree tear	14 (19%)	3 (23%)	0.49
Caesarean Section:			
Elective	21 (29%)	4 (31%)	0.56
Emergency	3 (4%)	0 (%)	0.61
Post-partum haemorrhage for atonic uterus	8 (11%)	2 (15%)	0.46
Neonatal Respiratory distress	5 (7%)	1 (8%)	0.64
	**Mean ± SD**	**Mean ± SD**	
Gestational Age at birth, weeks	39.1 (± 1.3)	39.6 (± 0.8)	0.08
Birth weight, kilograms	3.46 (± 0.49)	3.43(± 0.29)	0.77
Apgar 5 min	8.9 (± 0.6)	8.8 (± 0.6)	0.63

### Acceptability and feasibility of OGTT and CGM

Women reported CGM to be significantly more acceptable than OGTT (81% vs 27% 5/5 general acceptability rate, *p* < 0.001). One participant had uncontrollable nausea and vomiting, which she had also experienced with OGTT during her previous pregnancy. Her OGTT had to be stopped after the first hour, with only the first two blood glucose values being considered for diagnostic purposes by the treating team.

In the free comments’ section of the questionnaire on CGM acceptability, the most frequently reported issue (*n* = 10) was difficulty with keeping track of diet and exercise due to the requested time commitment and malfunctioning of the app.

### TRS and CGM parameters

One outlier for TRS was identified and removed (not included in the analysis as per Fig. 1) for a patient with a score deemed extreme compared to the rest of the cohort. This was due to the patient having selected “6 + servings/ day” for beef consumption, driving up the OR for iron and total red meat serving [12].

In the total cohort, the difference between 7 and 3 days of CGM data was significant (all *p* < 0.001) for sensor mean (4.1 ± 0.4 vs 3.9 ± 0.4 mmol/L), max value (7.3 ± 0.9 vs 6.8 ± 0.1 mmol/L), TIR during the day (81.8 ± 14.4% vs 72.5 ± 21.1%), and TIR at night (63.9 ± 26.5% vs 54.5 ± 28.8%). CV and MODD were significantly but only slightly higher when considering 3 vs 7 days of CGM (0.21 ± 0.1 vs 0.22 ± 0.04 and 0.80 ± 0.16 vs 0.76 ± 0.1 respectively). The difference between 3 and 7 days of CGM monitoring was not significant for SD, Min Value and MAGE.

Table 4 illustrates the differences in terms of TRS and CGM parameters (with both 3 and 7 days of monitoring considered) between women classified as NGT and GDM. No statistically significant differences were found. Women in the GDM group had higher TRS, CGMSV, SD, CV, MAGE and MODD and lower TIR and mean glucose values, both when 3 and 7 days were considered.

**Table 4:** TRS and CGM parameters of 3 and 7 days in NGT versus GDM

	**NGT (** ***n*** ** = 74)** **Median (IQR)**	**GDM (** ***n*** ** = 13)** **Median (IQR)**	***p*** **-value**
TRS	0.59 (0.69)	0.61 (0.41)	0.94
CGMSV			
- 3 days	3.83 (0.72)	3.91 (0.78)	0.95
- 7 days	4.14 (0.65)	4.33 (0.67)	0.55
TIR			
- 3 days	42.6% (33.6)	31.2% (10.5)	0.13
- 7 days	75.8% (34.6)	74.9% (29.5)	0.28
	**Mean ± SD**	**Mean ± SD**	
Mean			
- 3 days	3.96 ± 0.45	3.90 ± 0.40	0.67
- 7 days	4.16 ± 0.41	4.13 ± 0.43	0.85
SD			
- 3 days	0.85 ± 0.17	0.86 ± 0.14	0.84
- 7 days	0.85 ± 0.16	0.91 ± 0.19	0.27
CV			
- 3 days	0.21 ± 0.05	0.22 ± 0.04	0.69
- 7 days	0.20 ± 0.41	0.22 ± 0.46	0.24
MAGE			
- 3 days	2.00 ± 0.43	2.13 ± 0.36	0.27
- 7 days	2.01 ± 0.39	2.20 ± 0.46	0.18
MODD			
- 3 days	0.80 ± 0.16	0.81 ± 0.14	0.95
- 7 days	0.75 ± 0.13	0.81 ± 0.14	0.24

*TRS* Total risk factors score, *CGMSV* Continuous glucose monitoring score of variability, *TBR* Time below range, *TAR* Time above range, *SD* Standard deviation, *CV* Coefficient variation, *MAGE* Mean amplitude of glycaemic excursion, *MODD* Mean of daily differences

### Triangulation

**Fig. 2:**
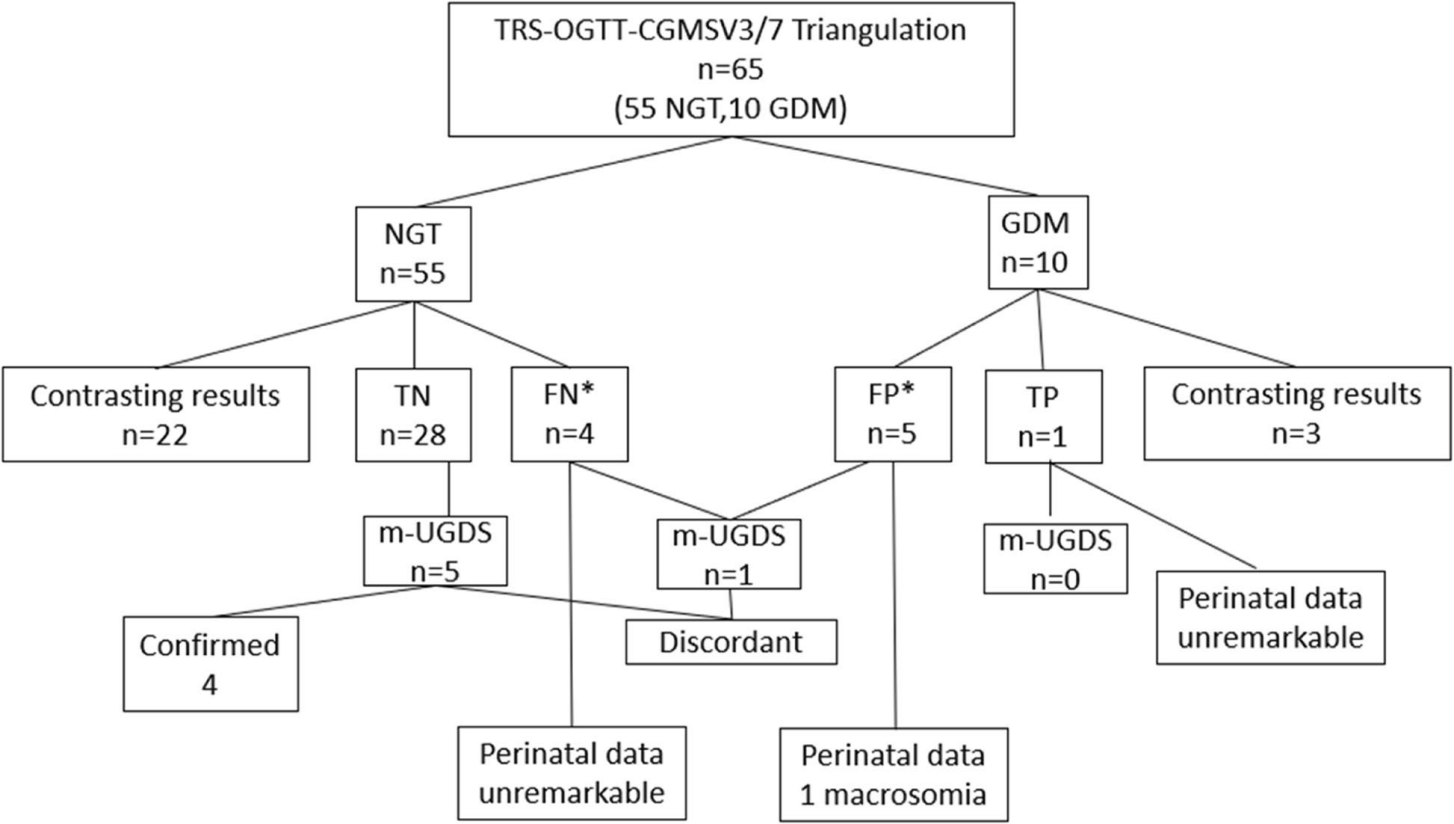
OGTT, TRS and CGMSV Triangulation TRS = Total Risk Score, OGTT = Oral Glucose Tolerance Test, CGMSV = Continuous Glucose Monitoring Score of Variability, NGT = Normal Glucose tolerance Test, GDM = Gestational Diabetes Mellitus, TN = True Negative, FN = False Negative, FP = False Positive, TP = True Positive, m-UGDS = modified Ultrasound Gestational Diabetes Score. Perinatal data: macrosomia (> 4.5 kg), hypoglycaemia, respiratory distress. *one additional case suggested by CGMSV3 only

The maximum TRS in the NGT population was 0.82 and the minimum score in the GDM population was 0.56. Therefore, the cut off value was determined to be 0.69; those above this value was considered to be at high-risk of GDM. Similarly, the maximum CGMSV in the NGT population was 5.52 for 3 days and 5.54 for 7 days and the minimum score in the GDM population was 2.85 for 3 days and 3.41 for 7 days. Therefore, the cut off value for high-risk from CGMSV was determined to be 4.18 for 3 days and 4.47 for 7 days. Triangulation of TRS-OGTT with concordant CGMSV3 and CGMSV7 (*n* = 63/65 = 97%) is outlined in Fig. 2.

Nine potential misdiagnoses of the OGTT were suggested by triangulating results of the CGMSV3 and CGMSV7 with TRS: five ‘false positive’ (positive OGTT but TRS and CGMSV 3/7 all below the cut-off) and four’false negative’ diagnoses (negative OGTT with TRS and CGMSV 3/7 all above the cut off). CGMSV3 suggested two additional misdiagnoses: one false positive (being below the cut-off in a GDM woman as opposed to CGMSV7) and one false negative (being above the cut-off in an NGT woman as opposed to CGMSV7).

Three of the twenty-five patients who underwent the ultrasound had an m-UGDS > 3 (12%). Adding UGDS fortified the true negative diagnosis (4 cases confirmed as not having GDM features) but not the potential misdiagnosis suggested by CGMSV3 (m-UGDS discordant in 1/6 patients considered false positive and 1/5 considered false negative who had been scanned). The analysis of outcomes in terms of macrosomia, respiratory distress and hypoglycaemia was additionally discordant. None of the five women considered to be false negative and the one considered true positive had any of the considered outcomes, whereas one of the six women considered false positive had a macrosomic newborn.

Table 5 shows the difference in TRS and CGMSV as well as the CGM parameters described above in the ‘NGT by triangulation’ group (including the false positives OGTT as well as the true negatives) with’GDM by triangulation’ group (including the false negative OGTT and the true positive) when considering CGMSV3 for triangulation.

**Table 5:** TRS and CGM parameters of 3 and 7 days in NGT and GDM by triangulation

	**NGT by triangulation** **(** ***n*** ** = 34)** **Median (IR)**	**GDM by triangulation** **(** ***n*** ** = 6)** **Median (IR)**	***p*** **-value**
**TRS**	**0.60 (0.07)**	**0.72 (0.07)**	** < 0.001**
CGMSV			
**- 3 days**	**3.74 (0.63)**	**4.45 (0.23)**	** < 0.001**
**- 7 days**	**3.99 (0.63)**	**4.66 (0.63)**	** < 0.001**
TIR			
- 3 days	40.71% (16.69)	39.32% (56.71)	0.625
- 7 days	70.80% (33.1)	70.1% (40.70)	0.571
	Mean ± SD	Mean ± SD	
Mean			
- 3 days	3.88 ± 0.40	4.12 ± 0.43	0.24
**- 7 days**	**3.86 ± 0.90**	**4.34 ± 0.35**	**0.03**
SD			
**- 3 days**	**0.78 ± 0.11**	**1.04 ± 0.11**	**0.01**
**- 7 days**	**0.81 ± 0.11**	**1.06 ± 0.21**	**0.03**
CV			
- 3 days	0.20 ± 0.03	0.26 ± 0.05	0.06
**- 7 days**	**0.20 ± 0.03**	**0.25 ± 0.06**	**0.103**
MAGE			
**- 3 days**	**1.87 ± 0.33**	**2.52 ± 0.34**	**0.01**
**- 7 days**	**1.93 ± 0.31**	**2.51 ± 0.45**	**0.02**
MODD			
- 3 days	0.76 ± 0.13	0.91 ± 0.16	0.07
**- 7 days**	**0.73 ± 0.11**	**0.92 ± 0.13**	**0.02**

*TRS* Total risk factors score, *CGMSV* Continuous glucose monitoring score of variability, *TBR* Time below range, *TAR* Time above range, *SD* Standard deviation, *CV* Coefficient variation, *MAGE* Mean amplitude of glycaemic excursion, *MODD* Mean of daily differences

Women defined as NGT by triangulation had significantly lower TRS, CGMSV, SD and MAGE than those considered GDM, both when 3 and 7 days of CGM data were considered. No significant difference was found for TIR.

## Discussion

To the best of our knowledge, this is the first study to assess the Freestyle Libre PRO for GDM diagnosis based on but not exclusive to the OGTT results. As expected, GDM women were more likely to have family history of diabetes mellitus and higher OGTT values [1, 11]. No significant difference was found in terms of demographics and perinatal outcomes. This could be due to the small sample size of the GDM group, but also to the non-reliable classification of glycemic metabolism offered by the OGTT.

Triangulation of OGTT results with CGM data, combined in the CGMSV3, and a comprehensive list of risk factors (TRS), suggested eleven potential misdiagnoses of the OGTT. The results of previous studies demonstrate the potential for CGM to unmask OGTT misdiagnosis [8–10]. In a study by Tartaglione et al., 33 of 53 women classified as NGT with the OGTT were then found to have blood glucose levels above or below the recommended thresholds at CGM and managed with one week of self-blood glucose monitoring and diet [10]. Twelve of these women ended up requiring insulin [10]. As in our study, Tartaglione et al. found no difference in average daily glucose, time spent in the different ranges and maternal and fetal outcomes between GDM and NGT [10]. In 2009 Hijazi found dysglycaemia with CGM in 2 of 9 OGTT negative patients [8]. A study by Milln on 28 women (20 GDM, 8 controls) reported instead potential false positives of the OGTT, with CGM glucose variability of women classified as having GDM being not different from those having a negative OGTT result once at home [9].

Our group has conducted preliminary studies on more than 80 patients using the Medtronic iPro2 CGM device and in an initial cohort of twenty-one women recruited in this pilot study (*n* = 21) [11, 12]. In the Medtronic pilot study, CGM was found to be safe and acceptable by the recruited pregnant women, with CGM values correlating well with 1-h (*p* = 0.003) and 2-h OGTT values (*p* = 0.004), and uncovering glycaemic variability that OGTT could not detect [11]. However, some women complained of irritation due to the overlying tape on their already sensitive abdomen, whilst others commented that they would prefer not to have daily finger pricking for calibration of the CGM device [11]. Our group proposed Abbott’s Freestyle Libre 2 CGM device to be more tolerable for pregnant patients, being wearable on the arm and not requiring finger pricking for calibration. We therefore sought advice from the Australian TGA who subsequently approved use of the FreeStyle Libre PRO for this study. The Freestyle Libre 2 CGM was reported as highly acceptable for GDM diagnosis by participating women, significantly more than the OGTT and with increased acceptability compared to the Medtronic Ipro2 pilot study [11].

During our recruitment, the woman suspending the OGTT after 1 h due to uncontrollable nausea and vomiting underscored the low acceptability of the OGTT deeply impacting completion rates, as previously reported in an Australian study [28].

The main disadvantage of our protocol was identified by women as having to keep track of diet and physical activity. Women also stated that they would have preferred a shorter CGM wearing period of three days. For this reason, a comparison between the first 3 days and the total period of CGM data was performed, showing significant differences for some CGM parameters only with contrasting results (e.g. higher distribution parameters (mean, max value) but lower variability (CV and MODD) and higher time in range (both daytime and night-time). No difference was found for the remaining parameters of distribution (min value, SD) and variability (MAGE). The variation in CGM3 and CGM7 parameters regarding OGTT diagnoses of GDM or NGT was similar.

The concept of triangulation is based on observing a phenomenon from different perspectives to fully comprehend it, adding a new frame of reference to consolidate the evaluation [29]. Triangulation with both well-established (e.g. family history of diabetes mellitus, age, BMI) and newly identified risk factors (diet composition, physical activity, season of conception, use of assisted reproductive technologies) in our cohort suggested OGTT misdiagnosis. This confirms the findings of our recent study on the development of an online questionnaire to recruit women at high and low risk of developing GDM, where triangulation analysis suggested six (13%) misdiagnoses (one false positive and five false negative cases) when both TRS and CGMSV resulted discordant with OGTT [12].

Considering 3 versus 7 days of CGM data resulted in conflicting differences regarding distribution/variability/time in range parameters, (e.g. better distribution but worse variability) suggesting that neither of the two timeframe performs better than the other in identifying a clear pattern of good/poor glycaemic control. This is reflected by the fact that at the triangulation analysis the results of CGMSV3 and CGMSV7 were concordant in 97% of the cases.

The additional two misdiagnosis cases (one false positive and one false negative) suggested by CGMSV3 compared to CGMSV7 favour its use for an initial screening phase. Evaluation of TRS and CGM data differences between women considered as NGT (true negatives and false positives) versus those considered GDM (true positives and false negatives) with triangulation adopting CGMSV3 highlighted significantly higher TRS as well as distribution (SD) and variability (MAGE) parameters in the GDM group. This result underlines the potential of CGM and triangulation in classifying glucose dysmetabolism of new onset in pregnancy. Adopting 3 days of CGM monitoring as a first step for GDM screening could represent a good compromise to increase acceptability whilst retaining diagnostic ability.

In the subgroup of 25 women who underwent an ultrasound, m-UGDS reinforced the true negative diagnosis but contrasted with the triangulation in one case considered false positive and one case considered false negative.

### Strengths, limitations and future directions

This pilot study reinforces the potential role of CGM in unmasking OGTT misdiagnosis and introduces the role of triangulation in aiding development of a new GDM screening tool when OGTT remains the ‘gold-standard’. Patients found CGM to be acceptable for GDM diagnosis, although suggested that the protocol could improve with a multistage approach, not encompassing diet and physical activity tracking during the screening phase. Only CGM data with complete acquisition (96 readings a day for 7 days of monitoring period) was included in our analysis to maximise its accuracy. Data collected with this pilot study on diet and training sessions are not reported in this manuscript. Our group is currently working on automating lifestyle data analysis independent of and in correlation with CGM data to allow for a more comprehensive and expedited evaluation of glucose metabolism in the everyday setting.

Our modified ultrasound score (m-UGDS) was evaluated in a small subgroup only given the delayed recruitment due to the COVID-19 pandemic. The results of this analysis need to be verified in further studies:we hope to adopt this method in larger future cohorts to verify its usefulness for triangulation. Only neonatal hypoglycaemia, macrosomia and respiratory distress were evaluated in terms of perinatal outcomes, with limited impact on the triangulation. An extended and systematic evaluation of perinatal outcomes as well as biomarkers, potentially combined in a score, could improve the triangulation. All the cut-off scores used for triangulation were based on the maximum and minimum values observed in the NGT and GDM group of this pilot study, limiting the comparison with our previous studies. The expansion of data acquisition at a multicentre level could permit the development of cut-offs based on and applicable to different settings, allowing for more reliable comparison of results. Based on the OR for macrosomia (> 4 kg), respiratory distress, preterm delivery and elective/emergency caesarean section reported in a recent meta-analysis for GDM women not using insulin, considering a relative precision of 50%, confidence level of 95%, and the rates of these outcomes resulted in this pilot study, a minimum sample size of 243 is required to explore the correlation of CGM data with at least one of these outcomes (caesarean section) (Additional file 4). To examine all these perinatal outcomes, at least 1041 patients is required [16].

The recent adaptation from WHO ASSURED to RE-ASSURED criteria, including now ‘Real-time-connectivity’, and ‘Ease-of-specimen-collection’ underlines the importance of investing in and expanding the promising potential of CGM as a screening test for GDM [30]. CGM fits well with both of these newly adapted criteria, and could allow for a more minimally invasive, remotely visualised and realistic picture of daily glycaemic control than the one represented by the OGTT.

## Conclusions

Freestyle Libre PRO 2 is an acceptable and feasible tool for CGM diagnosis. Future research on larger cohorts of patients considering additional biomarkers and multicentre-based scores is warranted to assess the use of CGM for the diagnosis of CGM on a broader scale and develop a triangulation system applicable to the general population of pregnant women in Australia. The realization of a multistage CGM diagnostic test for GDM could improve its acceptability and patients’ compliance as well as “inform in real-time, strengthen the efficiency of health care systems and improve patient outcomes” [30].

## Supplementary Information

**Additional file 1.** 

**Additional file 2.** 

**Additional file 3.** 

**Additional file 4.**

## Data Availability

The data that support the findings of this study are not publicly available due to their containing information that could compromise the privacy of research participants but are available from the corresponding author in a de-identified manner upon reasonable request.

## Abbreviations

BMI: Body Mass Index
CGM: Continuous Glucose Monitoring
CGMSV: Continuous Glucose Monitoring Score of Variability
CV: Coefficient of Variation
GDM: Gestational Diabetes Mellitus
GWG: Gestational Weight Gain
IADPSG: International Association of the Diabetes in Pregnancy Study Groups
MAGE: Mean Amplitude of Glycaemic Excursions
MODD: Mean of Daily Differences
NGT: Normal Glucose Tolerance
OGTT: Oral Glucose Tolerance Test
PCOS: Polycystic Ovary Syndrome
SD: Standard Deviation
TAR: Time Above Range
TBR: Time Below Range
TIR: Time in Range
T2DM: Type 2 Diabetes Mellitus
TRS: Total Risk Score
UGDS: Ultrasound Gestational Diabetes Score
m-UGDS: Modified UGDS
WHO: World Health Organization
