# Supplementary material for: A new continuous glucose monitor for the diagnosis of gestational diabetes mellitus: a pilot study
Source: BMC Pregnancy Childbirth. 2023 Mar 18;23:186. doi: 10.1186/s12884-023-05496-7 (PMC10023314; doi:10.1186/s12884-023-05496-7)
Supplement: Supplementary file 1 — Additional file 1. [file 12884_2023_5496_MOESM1_ESM.pdf]

Additional File 1. Risk factors questionnaire

How old are you? __________________________________

Do you have a family history of type 2 diabetes and/ or GDM? ◯ No ◯ Yes

If yes, what is their relation to you? (check all that apply)

☐ Cousin/ distant relative ☐ Mother-T2DM ☐ Father- T2DM ☐ Sibling ☐ Mother- GDM

Have you previously been diagnosed with GDM? ◯ No ◯ Yes

Have you previously given birth to a baby weighing >4.5kg? ◯ No ◯ Yes

Do you identify as Aboriginal or Torres Straight Islander? ◯ No ◯ Yes

Are you from any of the following backgrounds: Chinese, Southeast Asian, Middle Eastern or Indian? ◯ No ◯ Yes

If yes, where do you identify as?

◯ Hispanic ◯ South American ◯ Middle Eastern ◯ South Asian ◯ Chinese

What was your pre-pregnancy BMI?

◯ unknown ◯ < 18.5 ◯ 18.5-24.9 ◯ 25-29.9 ◯ >29.9

How much weight did you gain in your first trimester? (up to 14 weeks gestation)

◯ Not sure ◯ < 3.78kg ◯ 3.24-5.60kg ◯ >5.61kg

Have you been diagnosed with polycystic ovary disease (PCOS)? ◯ No ◯ Yes

Did you require the use of assisted reproductive technologies (IVF, ICSI) or was the pregnancy spontaneous? ◯ No ◯ Yes

If yes, what type did you use?

◯ Intracytoplasmic Sperm Injection (ICSI) ◯ In-Vitro Fertilisation (IVF)

Do you currently, or have you previously taken anti-depressant medication? ◯ No ◯ Yes

If yes, do/ did you take either of the following two medications? ◯ No ◯ Venlafaxine ◯ Amitriptyline

If yes, have you taken more than two antidepressant medications at once? ◯ No ◯ Yes

What season did you conceive in?

◯ Winter ◯ Spring ◯ Summer
◯ Autumn

Did you participate in any of the following activities on a regular basis during the year before you became pregnant? (check all that apply)

☐ Walking
☐ Swimming
☐ Jogging
☐ Weightlifting
☐ Dance/aerobics
☐ Bicycling
☐ Hiking
☐ Yoga
☐ Other activity

For walking, how many times per week? __________________________________

For walking, how many months did you regularly participate in this activity? __________________________________

For walking, how much time did you spend at the activity per episode? (in minutes) __________________________________

For swimming, how many times per week? __________________________________

For swimming, how many months did you regularly participate in this activity? __________________________________

For swimming, how much time did you spend at the activity per episode? (in minutes) __________________________________

For jogging, how many times per week? __________________________________

For jogging, how many months did you regularly participate in this activity? __________________________________

For jogging, how much time did you spend at the activity per episode? (in minutes) __________________________________

For weightlifting, how many times per week? __________________________________

For weightlifting, how many months did you regularly participate in this activity? __________________________________

For weightlifting, how much time did you spend at the activity per episode? (in minutes) __________________________________

For dance or aerobics, how many times per week? __________________________________

For dance or aerobics, how many months did you regularly participate in this activity? __________________________________

For dance or aerobics, how much time did you spend at the activity per episode? (in minutes)	______________________
For bicycling, how many times per week?	______________________
For bicycling, how many months did you regularly participate in this activity?	______________________
For bicycling, how much time did you spend at the activity per episode? (in minutes)	______________________
For hiking, how many times per week?	______________________
For hiking, how many months did you regularly participate in this activity?	______________________
For hiking, how much time did you spend at the activity per episode? (in minutes)	______________________
For yoga, how many times per week?	______________________
For yoga, how many months did you regularly participate in this activity?	______________________
For yoga, how much time did you spend at the activity per episode? (in minutes)	______________________
For your other activity, how many times per week?	______________________
For your other activity, how many months did you regularly participate in this activity?	______________________
For your other activity, how much time did you spend at the activity per episode? (in minutes)	______________________

The following questions will ask about your dietary patterns leading up to your pregnancy. For each food listed, choose the circle indicating how often on average you consumed it BEFORE becoming pregnant.

	Never, or less than once per month	1-3 per month	1 per week	2-4 per week	5-6 per week	1 per day	2-3 per day	4-5 per day	6+ per day
Eggs (1)	○	○	○	○	○	○	○	○	○
Beef (1 serving, ~170g)	○	○	○	○	○	○	○	○	○
Lamb (1 serving, ~170g)	○	○	○	○	○	○	○	○	○
Pork (1 serving, ~170g)	○	○	○	○	○	○	○	○	○
Bacon (2 slices)	○	○	○	○	○	○	○	○	○
Processed meats, e.g. salami, sausages, bologna (1 serving)	○	○	○	○	○	○	○	○	○
Shellfish, e.g. shrimp, lobster (1 serving)	○	○	○	○	○	○	○	○	○
Scallops, oysters, clams, mussels (1 serving)	○	○	○	○	○	○	○	○	○

Did you change your diet after finding out you were pregnant/ during pregnancy? ○ No ○ Yes

If you answered yes to the previous question, please re-fill the circles out reflecting how much you consume the listed foods NOW.

	Never, or less than once per month	1-3 per month	1 per week	2-4 per week	5-6 per week	1 per day	2-3 per day	4-5 per day	6+ per day
Eggs (1)	○	○	○	○	○	○	○	○	○
Beef (1 serving, ~170g)	○	○	○	○	○	○	○	○	○
Lamb (1 serving, ~170g)	○	○	○	○	○	○	○	○	○
Pork (1 serving, ~170g)	○	○	○	○	○	○	○	○	○
Bacon (2 slices)	○	○	○	○	○	○	○	○	○
Processed meats, e.g. sausages, salami, bologna (1 serving)	○	○	○	○	○	○	○	○	○
Shellfish, e.g. crab, loyster, prawns (1 serving)	○	○	○	○	○	○	○	○	○
Scallops, oysters, clams, mussels (1 serving)	○	○	○	○	○	○	○	○	○

Thank you for taking the time to complete the main questionnaire. Please rate your experience with this survey and provide any comments that you may have, all comments are appreciated and accepted.

Did you have trouble accessing the questionnaire?

I had great difficulty accessing this questionnaire — The questionnaire was easy to access and I had no trouble

(Place a mark on the scale above)

Did you understand every question in this questionnaire?

I did not understand any question in this questionnaire — I understood every question in this questionnaire

(Place a mark on the scale above)

Did you find this questionnaire generally acceptable?

I found this questionnaire very unacceptable — I found this questionnaire very acceptable

(Place a mark on the scale above)

Would you recommend this form of screening for gestational diabetes to other pregnant women?

I would not recommend — I would recommend

(Place a mark on the scale above)

Do you have any additional comments that you would like to make? All suggestions and comments are welcome. Thank you.

____
