# Supplementary material for: A new continuous glucose monitor for the diagnosis of gestational diabetes mellitus: a pilot study
Source: BMC Pregnancy Childbirth. 2023 Mar 18;23:186. doi: 10.1186/s12884-023-05496-7 (PMC10023314; doi:10.1186/s12884-023-05496-7)
Supplement: Supplementary file 2 — Additional file 2. [file 12884_2023_5496_MOESM2_ESM.pdf]

Additional file 2. CGM and OGTT questionnaires

CGM acceptability questionnaire -

Please fill this questionnaire to help us understand how pregnant women find CGM.

*Required

1. Email *

2. Name and Surname *

3. Phone number *

4. Did you find the device generally acceptable (tolerable)? *

Mark only one oval.

	1	2	3	4	5	
I found CGM overall very unacceptable	⬭	⬭	⬭	⬭	⬭	I found CGM overall very acceptable

5. Could you please rate on the following scale the acceptability of the insertion of the device: *

Mark only one oval.

	1	2	3	4	5	
Insertion was very unacceptable	⬭	⬭	⬭	⬭	⬭	Insertion was very acceptable

6. Could you please rate on the following scale the acceptability of wearing the device: *

Mark only one oval.

	1	2	3	4	5	
The device was difficult to wear or caused significant problems	⬭	⬭	⬭	⬭	⬭	The device was easy to wear and caused no probl

7. Could you please rate on the following scale the acceptability of removal of the device: *

Mark only one oval.

	1	2	3	4	5	
Removal of the device was very unacceptable	⬭	⬭	⬭	⬭	⬭	Removal of the device was very acceptable

8. Would you recommend this form of testing for gestational diabetes to other pregnant women? *

Mark only one oval.

	1	2	3	4	5	
I would not recommend	◯	◯	◯	◯	◯	I would recommend this form of testing to other pregnant women

9. Please provide any further comments in the space below:

____

____

____

____

____

This content is neither created nor endorsed by Google.

Google Forms

OGTT acceptability questionnaire -

Please fill this questionnaire to help us understand how pregnant women find OGTT.

*Required

1. Email *

____

2. Name and Surname *

____

3. Phone number *

____

4. Did you find the OGTT generally acceptable (tolerable)? *

Mark only one oval.

	1	2	3	4	5	
I found the OGTT overall very unacceptable	◯	◯	◯	◯	◯	I found the OGTT overall very acceptable

5. Could you please rate on the following scale the acceptability of having to fast to undergo the test: *

Mark only one oval.

	1	2	3	4	5	
Fasting was very unacceptable	◯	◯	◯	◯	◯	Fasting was very acceptable

6. Could you please rate on the following scale the acceptability of the glucose beverage: *

Mark only one oval.

	1	2	3	4	5	
The glucose beverage was very unacceptable	◯	◯	◯	◯	◯	The glucose beverage was very acceptable

7. Could you please rate on the following scale the acceptability of blood collection: *

Mark only one oval.

	1	2	3	4	5	
Blood collection was annoying or caused significant problems	◯	◯	◯	◯	◯	Blood collection was not annoying and caused no p

8. Could you please rate on the following scale the acceptability of the time frame of the test, including the 2 hours waiting period: *

Mark only one oval.

	1	2	3	4	5	
The time frame was very unacceptable	◯	◯	◯	◯	◯	The time frame was very acceptable

9. Would you recommend this form of testing for gestational diabetes to other pregnant women? *

Mark only one oval.

	1	2	3	4	5	
I would not recommend it	◯	◯	◯	◯	◯	I would recommend this form of testing to other pregnant women

10. Please provide any further comments in the space below:

____
____
____
____
____

This content is neither created nor endorsed by Google.

Google Forms
