# Supplementary material for: A new continuous glucose monitor for the diagnosis of gestational diabetes mellitus: a pilot study
Source: BMC Pregnancy Childbirth. 2023 Mar 18;23:186. doi: 10.1186/s12884-023-05496-7 (PMC10023314; doi:10.1186/s12884-023-05496-7)
Supplement: Supplementary file 3 — Additional file 3. [file 12884_2023_5496_MOESM3_ESM.docx]

Additional file 3. Clinical data extracted

- Pregnancy complications
- Date of OGTT
- 0, 1, 2 hours glucose values at OGTT
- Date of delivery
- Labour details
- Mode of delivery
- Perineal tears and repair
- Blood loss and reason for post-partum haemorrhage
- Birthweight
- Gestational age at birth
- Macrosomia suspected
- Neonatal sex
- Apgar
- Neonatal complications
- Neonatal Admission
