# Supplementary material for: A new continuous glucose monitor for the diagnosis of gestational diabetes mellitus: a pilot study
Source: BMC Pregnancy Childbirth. 2023 Mar 18;23:186. doi: 10.1186/s12884-023-05496-7 (PMC10023314; doi:10.1186/s12884-023-05496-7)
Supplement: Supplementary file 4 — Additional file 4. [file 12884_2023_5496_MOESM4_ESM.docx]

Additional file 4. Calculation of sample size for future studies

| OUTCOME | OR (16) | NGT PREVALENCE | SAMPLE SIZE |
| --- | --- | --- | --- |
| Macrosomia (>4 kgs) | 1.7 | 10.81% (8/74) | 415 |
| Respiratory distress | 1.38 | 6.75% (5/74) | 697 |
| Preterm delivery | 1.51 | 4.05% (3/74) | 1041 |
| LSCS (elective and emergency) | 1.16 | 32% (24/74) | 243 |

LSCS=Low segment caesarean section NOTE= relative precision set to 50% and confidence interval set to 95%.
